# Notch signaling drives multiple myeloma induced osteoclastogenesis

**DOI:** 10.18632/oncotarget.2084

**Published:** 2014-06-09

**Authors:** Michela Colombo, Katja Thümmler, Leonardo Mirandola, Silvia Garavelli, Katia Todoerti, Luana Apicella, Elisa Lazzari, Marialuigia Lancellotti, Natalia Platonova, Moeed Akbar, Maurizio Chiriva-Internati, Richard Soutar, Antonino Neri, Carl S. Goodyear, Raffaella Chiaramonte

**Affiliations:** ^1^ Department of Health Sciences, Università degli Studi di Milano, Milano, Italy; ^2^ Institute of Infection, Immunity and Inflammation, College of Medical, Veterinary and Life Sciences, University of Glasgow, Glasgow, UK; ^3^ Department of Clinical Sciences and Community Health, Università degli Studi di Milano; Hematology, Fondazione Cà Granda IRCCS Policlinico, Milano, Italy; ^4^ Division of Hematology and Oncology, Texas Tech University Health Sciences Center and Southwest Cancer Treatment and Research Center, Lubbock, TX, USA; ^5^ Beatson West of Scotland Cancer Centre, Haemato-oncology Service, Gartnavel Hospital, Glasgow, UK; ^6^ Laboratory of Pre-Clinical and Translational Research, IRCCS-CROB, Referral Cancer Center of Basilicata, Rionero in Vulture, Italy

**Keywords:** Myeloma, Notch, Jagged, RANKL, bone disease

## Abstract

Multiple myeloma (MM) is closely associated with bone destruction. Once migrated to the bone marrow, MM cells unbalance bone formation and resorption via the recruitment and maturation of osteoclast precursors.

The Notch pathway plays a key role in different types of cancer and drives several biological processes relevant in MM, including cell localization within the bone marrow, proliferation, survival and pharmacological resistance.

Here we present evidences that MM can efficiently drive osteoclastogenesis by contemporaneously activating Notch signaling on tumor cells and osteoclasts through the aberrant expression of Notch ligands belonging to the Jagged family. Active Notch signaling in MM cells induces the secretion of the key osteoclastogenic factor, RANKL, which can be boosted in the presence of stromal cells. In turn, MM cells-derived RANKL causes the upregulation of its receptor, RANK, and Notch2 in pre-osteoclasts. Notch2 stimulates osteoclast differentiation by promoting autocrine RANKL signaling. Finally, MM cells through Jagged ligands expression can also activate Notch signaling in pre-osteoclast by direct contact.

Such synergism between tumor cells and pre-osteoclasts in MM-induced osteoclastogenesis can be disrupted by silencing tumor-derived Jagged1 and 2. These results make the Jagged ligands new promising therapeutic targets in MM to contrast bone disease and the associated co-morbidities.

## INTRODUCTION

Multiple myeloma (MM) is a malignant plasma cell (PC) disorder accounting for approximately 10% of all hematological cancers. Although recent advances in treatment, myeloma remains an incurable disease [1].

MM is associated with osteoclast-mediated bone destruction, and consequent osteoporosis, hypercalcaemia, bone pain and fractures. Altogether, bony lesions are observed in up to 80% of patients [1, 2].

Circulating malignant PCs are recruited into the bone marrow (BM) by a chemotactic gradient involving the SDF-1/CXCR4 axis [3, 4]. BM-infiltrating MM cells induce BM stromal cells to increase the production of osteogenic factors such as the receptor activator of NF-κB ligand (RANKL) and may directly contribute to increase the level of RANKL [5, 6] and other pro-osteoclastogenic chemokines [6-8]. These events alter the ratio between RANKL and its decoy receptor, osteoprotegerin, thus increasing osteoclast (OCL) formation [9].

OCLs are involved in supporting MM cell long-term survival, proliferation and drug resistance [10], and promote TGF-β release from the bone matrix, which plays a role in antagonizing patient's anti-tumor immune responses [11]. They also cooperate with MM cells to stimulate new vessels formation, which in turn are able to induce osteoclastogenesis, promoting a vicious circle that leads to MM progression and bone lesions [12].

The Notch family includes four transmembrane receptors (Notch1-4), which are activated by ligands belonging to two families, Jagged (Jagged1, 2) and Delta-like (Dll1,3, 4). Receptor engagement activates the ADAM/TACE and the γ-secretase complex, triggering two proteolytic cleavages and the release of the intracellular portion of Notch (ICN). ICN translocates to the nucleus and activates the CSL (CBF1, Suppressor of hairless, Lag-1) transcription factor [3].

The Notch pathway plays a critical role in cell-fate decision, tissue patterning and morphogenesis and is dysregulated in a variety of malignancies [13, 14] including those affecting T- [15-17] and B-cells [18-20]. Importantly, Notch receptors are expressed by MM cells, BM stromal cells (BMSCs), and OCLs. MM cells activate the Notch pathway due to the over-expression of Jagged1 and Jagged2 ligands [21-23]. Jagged1 expression in malignant PCs arises upon progression from monoclonal gammopathy of undetermined significance (MGUS) to MM [23]. Jagged2 dysregulation [21, 24, 25] is an early event preceding MGUS, positively correlated with stage [24] and can be driven by epigenetic events [21] or overexpression of the ubiquitin-ligase Skeletrophin [25].

Functional evidences from this and other groups indicate that the active Notch signaling is involved in MM pathogenesis [3] and that its inhibition induces MM cell apoptosis, reduces drug resistance, and MM cell migration to the BM [4, 26]. The Notch pathway plays also a key role in bone tissue remodeling and skeletal development together with the NF-κB pathway [27-29].

Here, we provide experimental evidences that the Notch pathway drives MM-associated OCL development and bone destruction, which can be prevented by the inhibition of the dysregulated Jagged ligands on MM cells.

## RESULTS

### Notch signaling is required for myeloma-mediated osteoclastogenesis

The Notch pathway is essential in skeletal development and remodeling [27], since it drives OCL differentiation as reported by Fukushima et al. [28] and confirmed by our results which further indicate that Notch activity positively regulates *RANK* expression during osteoclastogenesis (Fig. S1).

These findings and the evidence that Notch plays a crucial role in MM cell biology [3] prompted us to investigate the contribution of Notch signaling in MM-induced osteoclastogenesis by analyzing: 1) MM cell osteoclastogenic property and 2) OCL differentiation.

**Figure 1:**
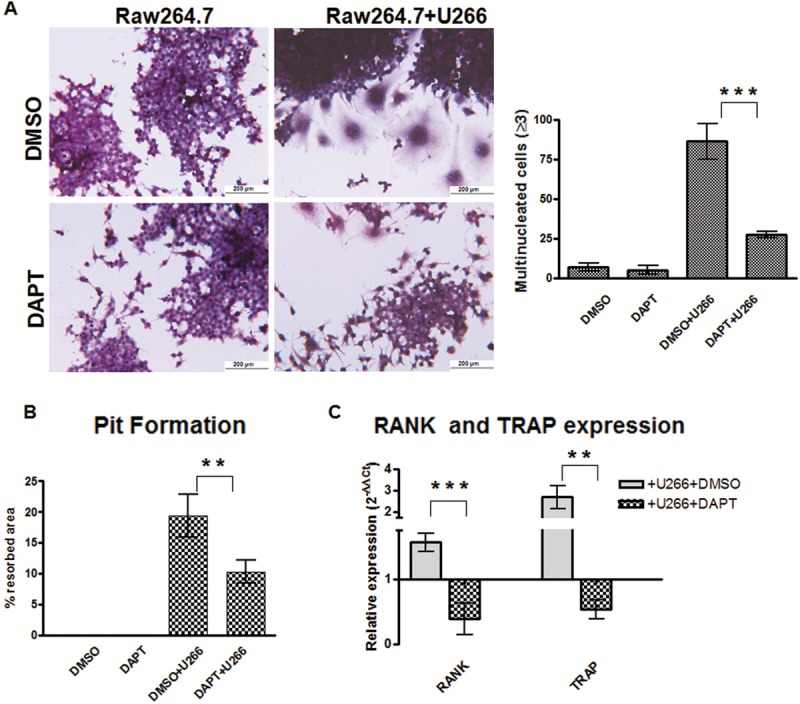
MM cells induce osteoclast differentiation in a Notch-dependent manner Co-culture system of Raw264.7 cells and U266 cells results in osteoclast differentiation which can be prevented by DAPT. (A) TRAP staining and enumeration of TRAP^+^/multinucleated cells in 7 days-single culture or co-cultures with or without DAPT. (B) Pit formation in the same cultures as (A) maintained for 10 days. (C) The relative gene expression of *TRAP* and *RANK* (normalized to GAPDH) in Raw264.7 + U266 cells ± DAPT was compared to Raw264.7 (DMSO) by the 2^−ΔΔCt^ formula. Graph shows the mean values ± SD. Two-tailed t-test confirmed statistically significant variations in the expression levels of *RANK* and *TRAP* when comparing co-cultures to single cultures in the presence of DMSO or DAPT; **= p <0.01, ***= p <0.001).

To investigate if the Notch pathway contributes to the process by which MM cells induce osteoclastogenesis, the U266 human MM cell line was co-cultured for 7 days with Raw264.7 cells with or without 50μM DAPT. U266 cells readily induced the formation of TRAP^+^/multinucleated Raw264.7 cells, which was significantly inhibited by DAPT (~70%). This finding indicated that the pro-osteoclastogenic ability of MM cells was dependent on active Notch signaling (Fig. 1A). Furthermore, Notch inhibition also impaired the osteolytic activity of OCLs generated in a 10 days Raw264.7/U266 co-culture assay (Fig. 1B). The need of an active Notch signaling in MM-induced osteoclastogenesis was further confirmed by the decrease in *TRAP* and *RANK* gene expression in Raw264.7 cells after DAPT treatment (Fig. 1C).

### MM cells induce OCLs formation by secreting RANKL in a Notch-dependent way

**Figure 2:**
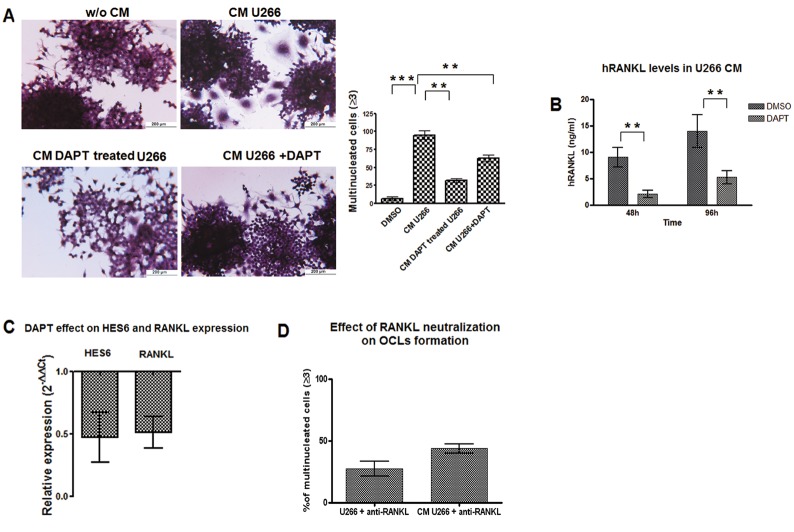
MM cells induce OCLs formation by a Notch-dependent release of *RANKL* To assess if MM cell osteoclastogenic property was dependent on Notch-driven secretion of soluble factors we evaluated the ability of U266-CM to induce OCL formation. (A) TRAP staining and enumeration of multinucleated Raw264.7 cells exposed to CM from U266 and additionally treated or not with DAPT, or exposed to CM obtained from DAPT-treated U266. Mean values ± SD are shown. Statistical analysis by ANOVA and Tukey test: **= p<0.01, ***= p <0.001. We also evaluated the ability of DAPT to inhibit RANKL expression in U266 cell line. (B) ELISA assay on RANKL protein released by U266 cell line in the CM after 48 and 96h DAPT treatment. SD were calculated from 3 independent experiments. Statistical analysis was performed using Two-tailed t-test: **= p<0.01. (C) qPCR measure of relative *RANKL* gene expression variation in DAPT-treated U266 cells compared to untreated cells, calculated by the 2^−ΔΔCt^ formula (as in Fig.1C); *HES6* gene expression variation confirmed DAPT treatment effectiveness. (D) U266 osteoclastogenic properties relies on the secreted RANKL: treatment with anti-RANKL antibody dramatically depletes OCL formation (TRAP^+^/multinucleated cells) in Raw264.7 cells cultured with U266 cells or U266-CM respect to the relative untreated controls (=100%). p<0.05 by ANOVA and Tukey post test for Raw264.7/U266/anti-RANKL vs Raw264.7/U266 and for Raw264.7/U266-CM/anti-RANKL vs Raw264.7/U266-CM.

We wondered if the ability of MM cell to induce Notch-dependent osteoclastogenesis was reliant upon the secretion of soluble factors. To test this hypothesis, we evaluated the osteoclastogenic property of U266 conditioned medium (CM). The contribution of U266-derived soluble factors was confirmed by the evidence that the addition of CM (20% V/V) to Raw264.7 cells for 7 days induced productive OCL differentiation. As expected, DAPT dramatically reduced CM-dependent osteoclastogenesis (Fig. 2A, CM U266 and CM U266 + DAPT), but more importantly the addition of CM from DAPT-treated U266 cells (Fig. 2A) was unable to induce OCL differentiation suggesting that the activation of Notch signaling was necessary for MM cells to produce osteoclastogenic soluble mediators.

Since Raw264.7 cell differentiation requires only RANKL stimulation, and MM cell ability to yield osteoclastogenic soluble factors depended on Notch activity, we hypothesized that U266 cells produced RANKL in a Notch-controlled manner. Indeed, U266 cells secreted 9.7 ng/ml and 14 ng/ml in 48h and 96h, respectively (Fig. 2B). DAPT treatment induced a significant decrease in RANKL transcript (Fig 2C) and secreted protein (Fig. 2B). DAPT effectiveness was confirmed by down-regulation of *HES6* expression. We confirmed that U266 cells pro-osteoclastogenic potential mainly depended on soluble RANKL released by these cells, indeed neutralizing RANKL antibody added to the co-culture system or U266 CM, dramatically reduced OCL differentiation (Fig. 2D).

### MM cell-derived RANKL promotes OCLs differentiation via Notch2 but not Notch1

**Figure 3:**
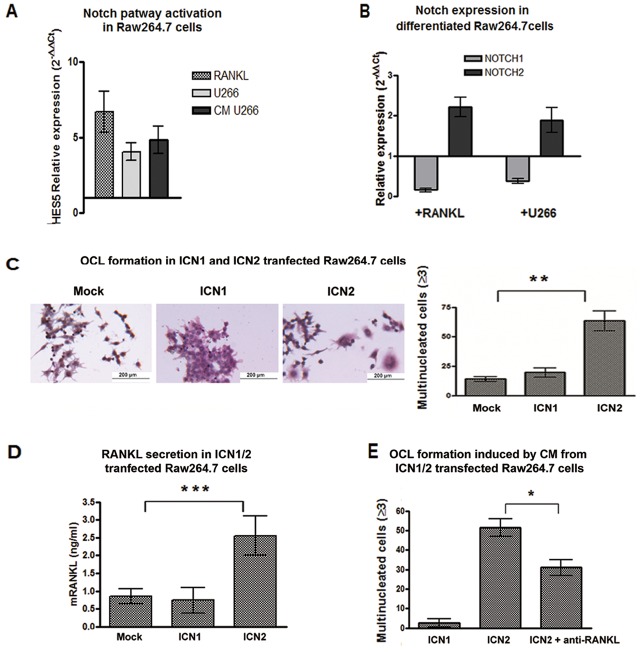
Notch2 is essential for OCL differentiation and drives RANKL secretion (A) U266 cells and U266-CM induce Notch activation in Raw264.7 cells: change in Notch activity level was measured as relative *HES5* gene expression variation in Raw264.7 cells cultured with U266 cells, U266-CM or RANKL compared to single cultured untreated cells (=1), and calculated by the 2^−ΔΔCt^ formula (as above). Mean values ± SD were shown. Two-tailed t-test confirmed statistically significant variation of Notch activity upon each treatment. (B) the relative gene expression of *Notch1* and *Notch2* (normalized to GAPDH) in Raw264.7 cells induced to differentiate in the presence of mRANKL or U266 cells compared to undifferentiated cells (2^−ΔΔCt^). (C) TRAP staining and enumeration of multinucleated cells in Raw264.7 cells 72h after electroporation with plasmids expressing Notch1 or Notch2. The graph shows the mean value ± SD. Statistical analysis by ANOVA and Tukey post-test; **= p <0.01 (D) ELISA for RANKL secretion in CM from transfected Raw264.7 cells. Mean values ± SD are shown. Statistical analysis by ANOVA and Tukey post-test (***=p <0.001). (E) Enumeration of TRAP^+^/multinucleated cells on Raw264.7 cells exposed to the CM from ICN1- or ICN2-transfected Raw264.7 cells, or the CM from ICN2-transfected cells with RANKL neutralizing antibody. Results were normalized to CM from mock cells (for ICN1- and ICN2-transfections) or mock cells + RANKL neutralizing antibody (only for CM from ICN2-transfected cells). Standard deviations were calculated from 3 independent experiments and statistical significance (ICN1 vs ICN2; ICN2 vs ICN2+anti-RANKL) was verified by Two-tailed t-test (*=p <0.05).

Since RANKL secretion seemed to be crucial in determining the osteoclastogenic property of MM cells, we focused on the outcome of RANKL stimulation on OCL progenitors. Basing on Duan and colleagues [30] RANKL stimulation resulted in Notch signaling activation in OCLs, therefore we wondered if U266 cells were able to trigger Notch signaling in Raw264.7 cells by releasing RANKL. At this purpose, Raw264.7 cells were cultured for 5 days with U266 cells, U266-CM (20% V/V) or mRANKL alone (50 ng/mL). In all conditions *HES5* transcript was up-regulated (Fig.3A), thus indicating that MM cells could trigger the osteoclastogenic Notch signaling in OCL precursors by releasing RANKL and did not necessarily need a direct interaction.

We wondered if the observed changes in Notch signaling could be due to a variation in the expression of Notch isoforms relevant in MM. Our results showed that OCL differentiation induced by RANKL or MM cells was associated to an increase in *Notch2* and a decrease in *Notch1* level (Fig. 3B), suggesting a different role for the two Notch isoforms during osteoclastogenesis. To address this issue, we analyzed the effect of the two Notch isoforms by transiently transfecting Raw264.7 cells with plasmids carrying ICN1 and ICN2. Increased expression and activity of Notch1 and 2 was confirmed by Western blot for their active forms (Fig.S2-panel A), and by RT-PCR for the Notch transcriptional target gene *HES5* (Fig.S2-panel B). Figure 3C shows that ICN2, but not ICN1, induced Raw264.7 cell osteoclastogenesis and increased *TRAP* expression 3 days after electroporation (Fig.S2-panel B). ICN2 stimulated the expression of RANKL in Raw264.7 cells, as confirmed by PCR (Fig.S2-panel B) and ELISA (Fig.3D). Moreover, the CM from ICN2-transfected Raw264.7 cells induced osteoclastogenesis when added to not-transfected Raw264.7 cells after 7 days (Fig.3E). To confirm that this effect was dependent on the increased *RANKL* production, a neutralizing anti-RANKL antibody was added to the CM from ICN2-transfected Raw264.7 cells. RANKL neutralization significantly inhibited the ability of CM to induce osteoclastogenesis (Fig.3E and Fig.S2-panel C).

### RANKL expression in CD138^+^ cells from MM patients is correlated with Notch pathway activation

**Figure 4:**
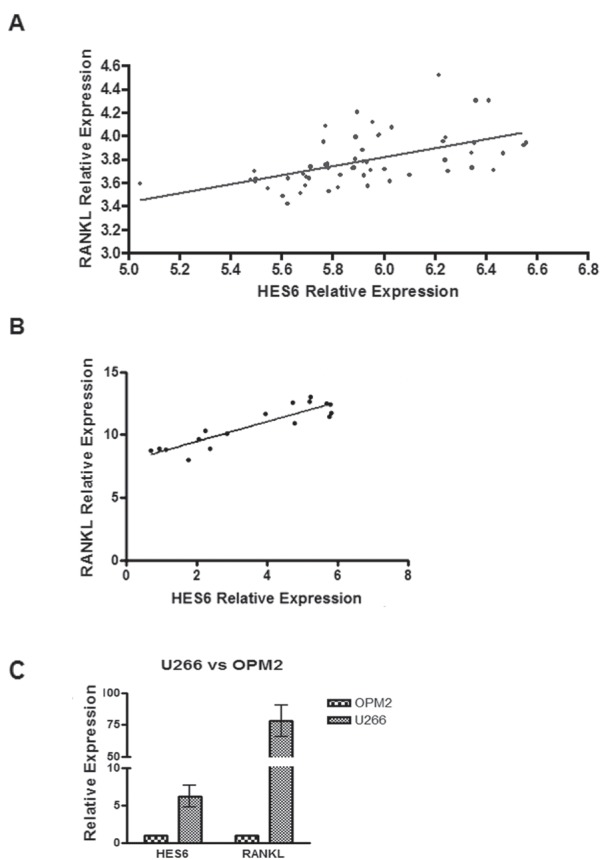
Notch pathway activation is correlated with RANKL expression levels in MM patients (A) Correlation of *RANKL* and *HES6* gene expression levels in purified PC samples from 55 MM patients profiled on Affymetrix Gene 1.0 ST array. Statistical analysis was performed with Pearson's rank correlation test (p= 2.6e-05). (B) qRT-PCR of *RANKL* and *HES6* genes on 17 MM patients. Gene expression is relative to GAPDH. Statistical analysis: Pearson's correlation coefficient=0,9087; p<0,0001. (C) qRT-PCR for HES6 and RANKL on U266 and OPM2 cell lines. Data are presented as the relative expression in U266 vs OPM2 (=1), calculated by the 2−^ΔΔCt^ formula (see above). Mean values ± SD of 3 independent experiments are shown.

To investigate if *RANKL* gene expression in MM cells could be associated with Notch pathway activation in MM patients, we evaluated *RANKL* and Notch target *HES6* gene expression profiles from a proprietary data set (GEO accession number: GSE 39925) of highly purified PC samples from 55 newly-diagnosed MM patients [31] profiled on Affymetrix Gene 1.0 ST array. Analysis revealed a significant correlation between the Notch target, *HES6*, and *RANKL* expression levels (Fig. 4A; Pearson's correlation coefficient=0.53439).

Furthermore, validation qRT-PCR on mRNA from 17 MM patients' CD138^+^ cells confirmed the correlation between *HES6* and *RANKL* transcript levels (Fig. 4B, Pearson's correlation coefficient=0,9087). Analogously, *HES6* level was higher in the high RANKL-expressing U266 cells in comparison to the low RANKL-expressing MM OPM2 cell line (Fig. 4C). These findings are consistent with the evidence that RANKL expression by MM cells depends upon Notch activity.

### Notch signaling inhibition reduces the osteoclastogenic potential of primary human MM cells

To confirm the crucial role of Notch signaling in MM-associated bone disease *ex vivo*, we inhibited the Notch signaling during the differentiation of primary human monocytes induced by addition of osteoclastogenic factors or by co-culturing with primary human MM cells.

**Figure 5:**
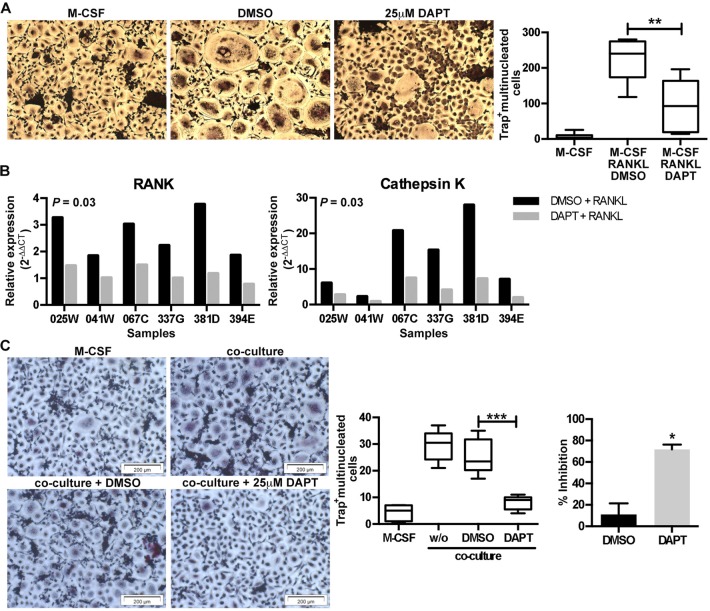
Inhibition of Notch signaling inhibits RANKL- and myeloma cell-induced osteoclastogenesis (A) Human CD14^+^ monocytes (n = 6) were stimulated with M-CSF or M-CSF plus RANKL in the absence (DMSO) or presence of DAPT (25μM). After 8 days the number of TRAP+ multinucleated cells (≥3 nuclei) was enumerated. Representative images are shown for each condition and a box & whisker plot, where the boxes represent the 25th to 75th percentiles, the lines within the boxes represent the median, and the lines outside the boxes represent the 5th and 95th percentiles, show the absolute number of TRAP+ multinucleated cells. **= p < 0.01 by a one-way ANOVA with Tukey's multiple comparison post-test. (B) RNA was extracted at day 3 and q-RT-PCR was performed to evaluate the level of *RANK* and *Cathepsin K* expression. Gene expression was normalized to B2M and the fold-change was calculated by qRT-PCR as reported above. Significance was determined by a Wilcoxon matched-pairs signed rank test. (C) Human CD14^+^ pre-osteoclasts were stimulated with M-CSF or M-CSF plus co-cultured with primary myeloma cells in the absence (DMSO) or presence of DAPT (25μM). After 7 days the number of TRAP+ multinucleated cells (≥3 nuclei) was enumerated. Representative images and a box & whisker plot, where the boxes represent the 25th to 75th percentiles, the lines within the boxes represent the median, and the lines outside the boxes represent the 5th and 95th percentiles, show the absolute number of TRAP+ multinucleated cells per image (n = 8) for one experiment. ***= p < 0.001 by a one-way ANOVA with Dunnett's multiple comparison post-test. This was repeated in 4 independent experiments and the % inhibition over the experiments is shown in the bar graph. *= p<0.05 by Mann-Whitney test.

Purified human CD14^+^ monocytes were cultured with M-CSF and RANKL in the presence of 25 μM DAPT for 8 days. DAPT significantly reduced RANKL-induced OCL differentiation (Fig. 5A) and suppressed the upregulation of the transcript levels of *RANK* and *Cathepsin K* at day 3 (Fig. 5B). At the same time, we performed co-cultures of human CD14^+^ monocytes with primary human CD138^+^ cells purified from myeloma patient BM aspirates. DAPT significantly inhibited the ability of myeloma cells to induce osteoclastogenesis (Fig. 5C), confirming the results in previously described Raw264.7/U266 co-cultures.

### MM-derived Jagged ligands promote OCL differentiation by activating Notch signaling on MM cells and pre-OCLs

**Figure 6:**
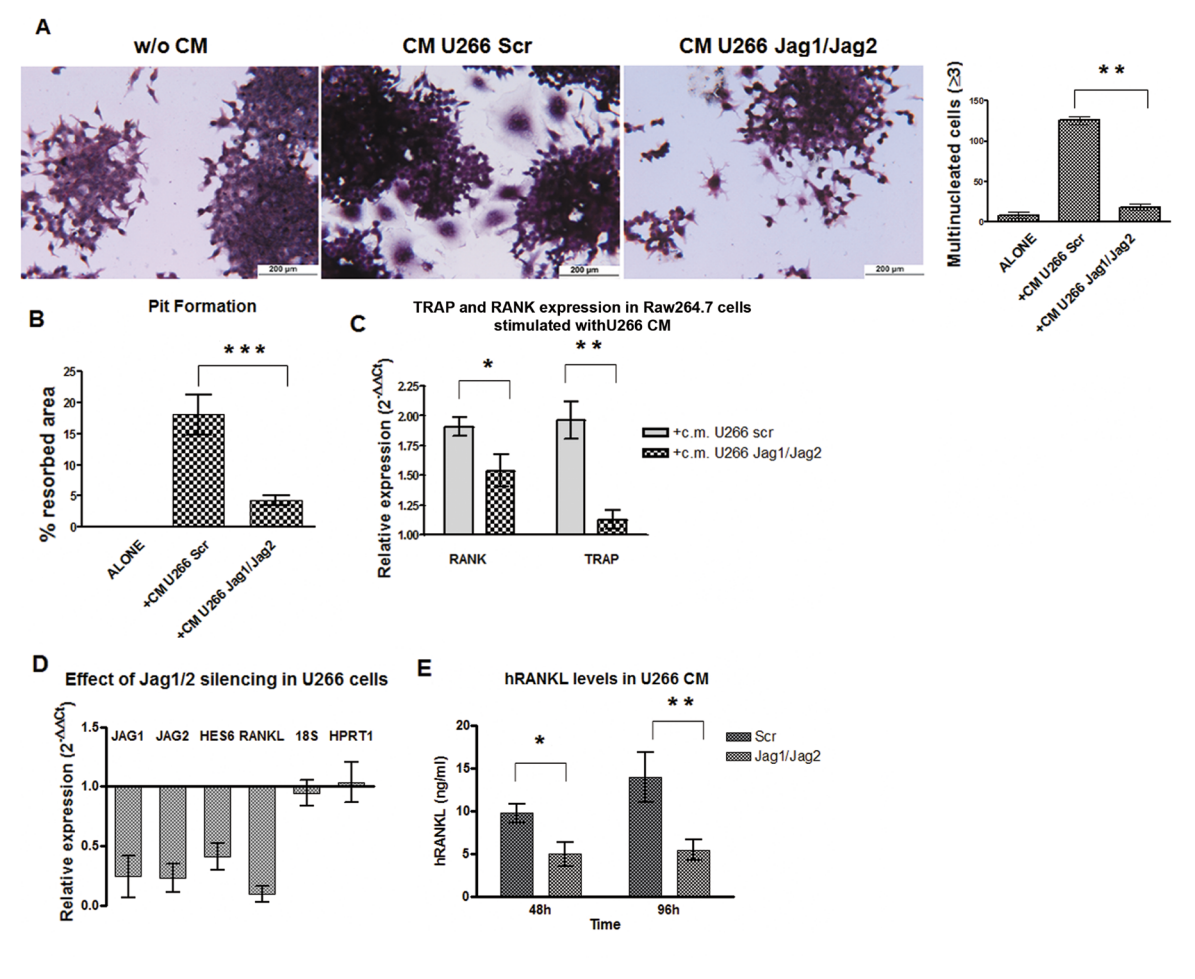
Jag1/2 silencing impairs MM cell osteoclastogenic ability To verify if U266-derived Jagged ligands affect the release of soluble osteoclastogenic factors, Raw264.7 cells were cultured in the presence of CM from J1/J2- or Scr-transfected U266 cell line. The osteoclastogenic ability of J1/J2-silenced U266 CM was abated as shown by: (A) TRAP staining and enumeration of TRAP^+^/multinucleated Raw264.7 cells and (B) Pit formation assay. Statistical analysis was performed using the ANOVA and Tukey test: ***= p <0.001; (C) *RANK* and *TRAP* genes upregulation is reduced in Raw264.7 cells cultured in the presence of J1/J2-silenced U266 CM compared to Scr-U266 CM. Data are presented as mean values ± SD of relative gene expression calculated by the 2^−ΔΔCt^ formula (see above). Two-tailed t-test confirmed statistically significant variations in the expression levels of *RANK* and *TRAP* induced by J1/J2- or Scr-transfected U266; *= p <0.05, **= p <0.01. Jagged ligands inhibition resulted in a reduced secretion of RANKL by MM cells. U266 cells were transfected with J1/J2 specific siRNAs or with the Scr control: (D) qRT-PCR of *RANKL* gene expression 48h post transfection in J1/J2-silenced U266 compared with Scr cells (2^−ΔΔCt^ formula); *Jagged1, Jagged2* and *HES6* were analyzed as control of pathway inhibition and *18s* and *HPRT1* as control of treatment specificity; (E) 48 and 96h post-silencing ELISA assay for RANKL secreted in the CM of J1/J2-silenced and Scr U266 cells. Statistical analysis was performed using Two-tailed t-test: *=p<0.05 **=p <0.01. SD were calculated from 3 independent experiments.

Notch pathway dysregulation in MM is mainly due to the alterations of two Notch ligands, Jagged1 and Jagged2. To test their contribution in MM-induced osteoclastogenesis, Raw264.7 were cultured for 7 days with the CM from U266 transfected with Jagged1 and Jagged2 siRNAs (J1/J2) or the corresponding scrambled siRNAs (Scr). J1/J2 silencing did impair the ability of U266 CM to promote the generation of osteolytically active TRAP^+^/multinucleated cells (Fig. 6A and 6B), and compromised the upregulation of *TRAP* and *RANK* expression in Raw264.7 (Fig. 6C). The effectiveness of J1/J2 silencing in U266 cells and the consequent Notch pathway inhibition were verified by qRT-PCR shown in Fig. 6D; two housekeeping genes (*18s* and *HPRT1*) were used as control of siRNAs specificity. qRT-PCR analysis revealed that the expression level of *RANKL* was significantly reduced in J1/J2-silenced U266 cells after 48h (Fig. 6D). This effect was associated with decreased expression of soluble RANKL in CM (Fig. 6E). These results further support the evidence that MM cells require Jagged-activated Notch to trigger OCL differentiation through the expression of RANKL.

**Figure 7:**
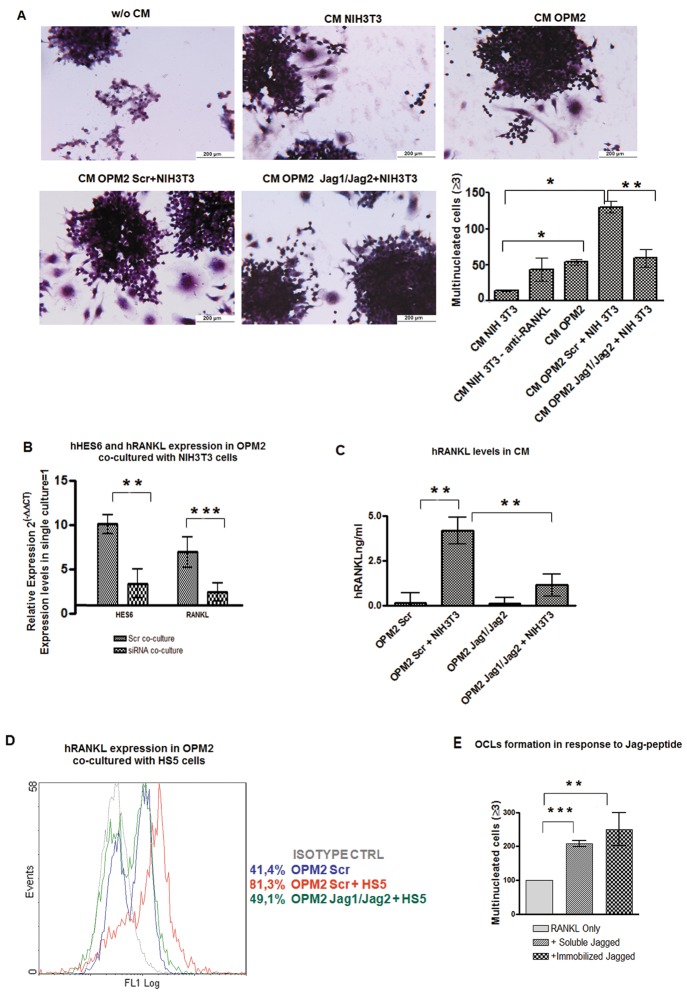
Stromal cells stimulate RANKL expression in MM cells in a Notch-dependent manner Interaction with stromal cells promotes osteoclastogenic potential of low-osteoclastogenic OPM2 cells by inducing RANKL secretion. J1/J2 silencing of OPM2 cells hampers stromal cells effect. (A) OCL formation by Raw264.7 cells cultured with CM from OPM2 cells, CM from NIH3T3 cells, or CM from OPM2 cells transfected with J1/J2- or Scr-siRNAs and co-cultured with NIH3T3. TRAP staining and mean value of counted OCLs ± SD are shown. Statistical analysis was performed using the ANOVA and Tukey test: *=p<0.05, **= p <0.01. (B) qRT-PCR for of *RANKL* and *HES6* genes expression in J1/J2- or Scr-transfected OPM2 co-cultured with NIH3T3 cells compared to Scr-transfected OPM2 alone (=1), calculated by the 2^−ΔΔCt^ formula. *HES6* as control of pathway activity. Mean values ± SD are shown. Statistical analysis by two-tailed t-test :**= p<0.01; ***=p<0.001. (C) ELISA assay on RANKL secreted by OPM2 cell line transfected with J1/J2 or Scr siRNAs and cultured alone or with NIH3T3 cells. Mean values ± SD are shown. Statistical analysis by one way ANOVA and Tukey post test: **= p<0.01. (D) Histograms display the levels of intracellular RANKL analyzed by flow cytometry in Scr-transfected OPM-2 in single culture (blue line) or in co-culture with HS5 cells (red line) and J1/J2-transfected cells co-cultured HS5 cells (green line); isotype-matched control (gray line). (E) To verify if MM cell-derived Jagged ligand directly contributes to OCL formation by interacting with pre-OCLs, Raw264.7 cells were induced to differentiate with 30ng/ml of mRANKL in the presence or absence of 0,5 ng/ml of soluble or immobilized Jagged. Graph shows the percentage of multinucleated cells in every culture normalized to mRANKL only treated cells (=100%). Error bars represent SD calculated out of 3 independent experiments. Statistical analysis was performed by ANOVA and Tukey test: **= p <0.01; ***= p <0.001

Finally, it is an accepted notion that not all primary MM cells and cell lines are able to secrete significant amounts of RANKL [32, 33], i.e. OPM2 cells express very low levels of RANKL (Fig.4C). To verify if the interaction with BMSCs could enhance the osteoclastogenic potential of low RANKL-expressing MM cells, we get advantage of a co-culture system including NIH3T3 fibroblasts as mimic for BM stromal cells. Interestingly, we found that CM obtained by co-culturing OPM2 with NIH3T3 cells had a greater pro-osteoclastogenic potential when compared to CM from OPM2 or NIH3T3 cells alone, while this effect was absent when J1/J2-silenced OPM2 cells were co-cultured with NIH3T3 cells (Fig. 7A). Accordingly, OPM2 co-cultured with NIH3T3 cells displayed increased RANKL and *HES6* gene expression (Fig. 7B) and secreted RANKL (Fig.7C). These effects were dramatically reduced upon J1/J2 silencing (Fig. 7B and C). To exclude any possible bias due to the use of a murine fibroblast cell line as mimic of BMSCs, the above results were confirmed by co-culturing MM cells with the human BMSC line HS5. Indeed, Fig. 7D shows that HS5 cells increased up to twofold RANKL expression in OPM2 cells. This effect was almost completely impaired by J1/J2 silencing. These results suggest a new mechanism by which BMSCs support MM-associated bone disease and indicated its dependency on the expression of Jagged ligands on MM cells.

Although the evidence that CM from MM cells is sufficient to drive osteoclastogenesis by upregulating Notch signaling in OCL progenitors, evidences from BM metastatic breast cancer cells showed that tumor-derived Jagged1 promoted OCLs differentiation by triggering Notch activation through a direct contact [34]. Therefore, we explored if also MM cells exploited Jagged ligands to directly activate Notch signaling in OCL precursors. To exclusively assess the level of Notch activation in pre-OCLs triggered by Jagged, we induced Raw264.7 differentiation (30 ng/mL RANKL) in the presence or the absence of Jagged1. We observed that stimulation with 0.5 μg/ml soluble or immobilized Jagged1 increased OCL differentiation (Fig. 7E) when cells were cultured in the presence of RANKL, while Jagged1 alone did not affect differentiation (data not shown). These findings indicate that the autonomous release of RANKL by tumor cells is fundamental for MM-induced osteoclastogenesis, and MM cell-derived Jagged can further boost OCL differentiation by directly triggering the osteoclastogenic Notch signaling in OCL precursors.

## DISCUSSION

MM is characterized by osteoporosis and bone lesions in almost 80% of patients [3]. Osteolysis not only affects patients quality of life, but also promotes tumor burden [10], angiogenesis [12], drug resistance [35, 36] and reduces the patient's anti-tumor immune response [3, 11], thereby contributing to the fatal outcome of MM. Therefore bone disease is a relevant issue in MM.

Notch has been proposed as a promising therapeutic target in MM [4, 26, 37]. Notch receptors and ligands are dysregulated in MM and positively correlate with clinical stage [21-25]. Notch signaling promotes MM cell proliferation, survival [4, 16, 37-40] and drug resistance [38, 41]. Recently, we have described that Notch signaling is involved in malignant PC localization at the BM by controlling the expression of the chemokine receptor CXCR4 [4]. A well-known effect of MM localization in the BM is the unbalance of the OCL/OBL ratio by increasing osteoclastogenesis and reducing OBL differentiation, finally resulting in bone disease. Interestingly, the Notch pathway is also determinant in skeletal development and remodeling [27, 28].

Based on these considerations, we investigated the role of Notch signaling in MM-induced osteoclastogenesis by: 1) confirming its outcome on OCL differentiation and 2) analyzing if Notch signaling dysregulation affects the osteoclastogenic potential of MM cells.

We confirmed that osteoclastogenesis needs an active Notch signaling by inhibiting Notch via DAPT on OCL precursors, the murine Raw264.7 monocyte cell line, or human monocytes from healthy donors. Interestingly, also MM-associated osteoclastogenesis required an active Notch signaling. Indeed, getting advantage of co-culture systems of MM cells and OCL progenitors (involving cell lines as well as primary cells), we observed that the inhibition of Notch signaling hinders the ability of MM cells to drive OCL differentiation. These findings raised the question if the observed anti-osteoclastogenic effect was simply due to Notch inhibition in OCLs or it could be also attributed to a reduced Notch signaling in MM cells.

**Figure 8:**
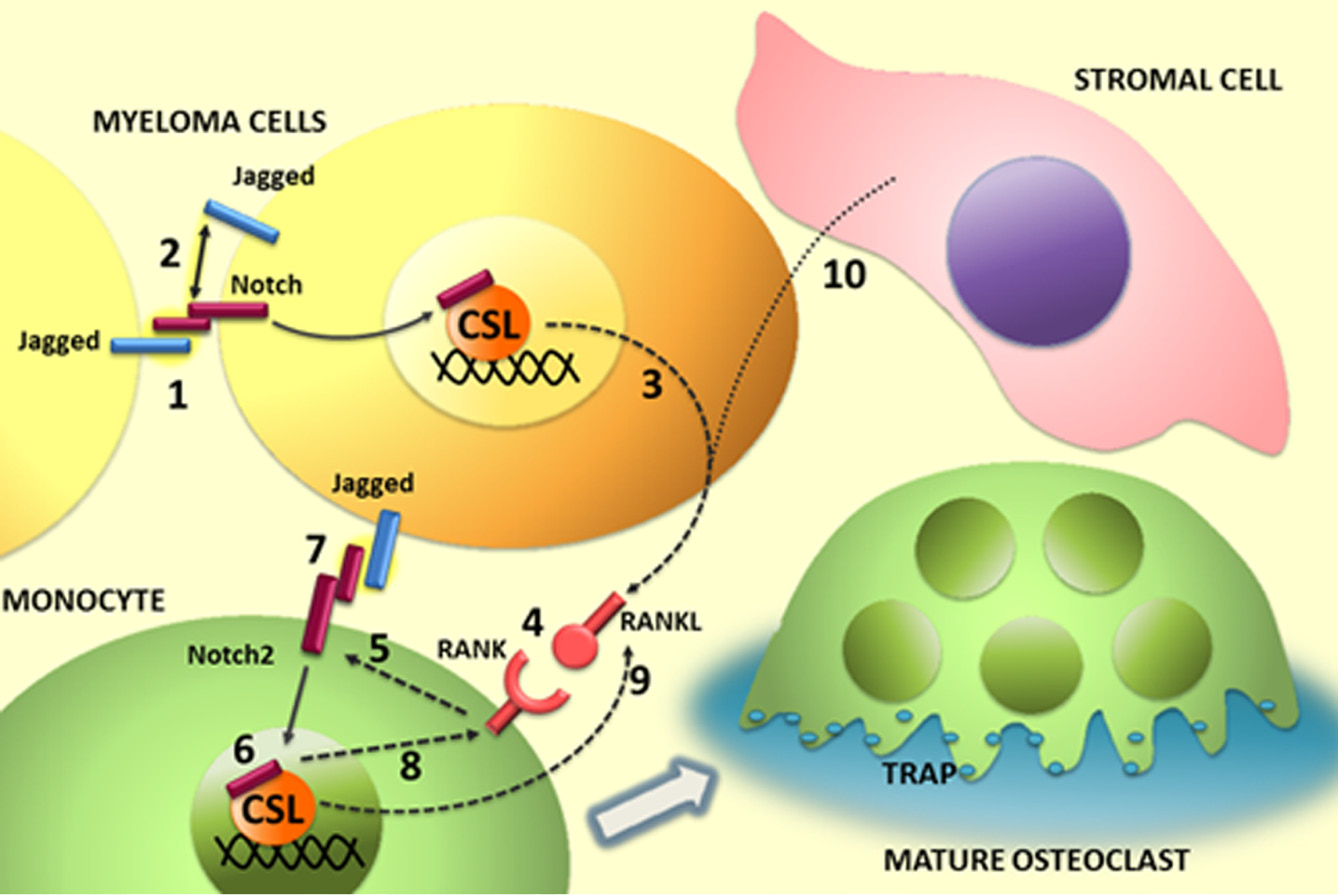
Illustration of the role of Notch ligands and receptors in MM-associated osteoclastogenis In MM cells Notch signaling can be activated in trans (1) by homotypic interaction between neighboring tumor cells or in cis through binding of ligands and receptors expressed by the same cell. Notch activation in MM cell results in (3) the autonomous production of the osteoclastogenic factor RANKL and the engagement of RANK on osteoclast progenitor (4). In turn, RANK signaling stimulates Notch2 gene expression (5) and its transcriptional activity (6). Jagged ligands on myeloma cell surface may contribute to enhance the osteoclastogenic process by Notch2 engagement and activation (7). The osteoclastogenic effect of Notch2 signaling results, at least in part, from the increased level of RANK (8) and secretion of RANKL (9). Stromal cells can enhance the osteoclastogenic potential of myeloma cells by stimulating their autonomous production of RANKL (10). This effect depends on Jagged ligands expressed by myeloma cells.

We wondered which could be the contribution of Notch signaling to MM cell osteoclastogenic potential and reasoned that the contemporaneous expression of Notch receptors and ligands could allow MM cells to autonomously activate Notch signaling as well as to trigger (via surface Jagged) the osteoclastogenic activity of Notch on neighboring pre-OCLs (as illustrated in Fig.8).

Concerning the first point, by using co-culture systems, we investigated if the endogenous Notch activation resulted in MM cell release of soluble osteoclastogenic factors. We demonstrated, for the first time, that the osteoclastogenic potential of MM cells depended on Notch signaling ability to induce the autonomous RANKL secretion (illustrated in Fig.8). Notch ability to drive MM cells pro-osteoclastogenic potential is mainly due to its capability to regulate RANKL secretion, since RANKL neutralization in Raw264.7 cells cultured with U266 or U266-CM impaired OCL formation.

Although our findings indicated that Notch activity can promote the osteoclastogenic potential of MM cells inducing the secretion of RANKL, not all primary MM cells or cell lines produce RANKL and are osteoclastogenic. Interestingly, we found that BMSCs were able to promote the osteoclastogenic potential of MM cells lacking osteoclastogenic properties. Indeed, low-osteoclastogenic OPM2 cells co-cultured with murine fibroblasts or human BMSCs strongly increased RANKL secretion and improved their ability to induce OCL formation. Remarkably, this effect required an active Notch signaling, since BMSCs could not enhance the osteoclastogenic potential of J1/J2-silenced OPM2 cells.

These findings provide further insight in the interaction between MM and BM microenvironment, suggesting that Notch signaling deregulation might be a key step in MM progression, which provides osteoclastogenic potential to MM cells by increasing their sensibility to stromal cells stimulation.

The evidence that the osteoclastogenic potential of MM cell depends on Notch activity, via the release of RANKL, represents an important change in the current view. The clinical relevance of these findings stems from the following evidences: 1) Notch activity (assessed as *HES6* gene expression) and *RANKL* expression are directly correlated in primary MM cells and in the differently osteoclastogenic MM cells lines (U266 and OPM2) used in this work; 2) the inhibition of Notch signaling hampers the pro-osteoclastogenic potential of primary MM cells; 3) RANKL expression in MM cells correlates with osteolytic bone disease [42, 43], and, accordingly, 4) RANKL targeting has been reported to prevent myeloma bone disease [44].

Our investigation on MM cells osteoclastogenic properties took in consideration also the effect of the direct contact of MM cells with OCL progenitors. We reasoned that dysregulated Jagged ligands expressed on MM cell surface [21-25] could engage Notch receptors on neighboring pre-OCLs, resulting in the direct activation of the osteoclastogenic Notch signaling. To assess if this direct interaction occurred, Raw264.7 cells were cultured with Jagged1. The evidence that Jagged-stimulated Raw264.7 cells doubled RANKL-induced OCL formation prompted us to conclude that MM exploits tumor-derived Jagged to engage Notch receptor in OCLs thus increasing RANKL osteoclastogenic effect. Therefore, BM-localized tumor cells may take advantage of Jagged ligands to promote OCL differentiation in two different ways: 1) by directly activating the osteoclastogenic Notch pathway in OCL progenitors and 2) inducing tumor cells to secrete RANKL autonomously or in response to BMSCs stimulation. Of note, while MM-osteoclastogenic potential is mainly based on RANKL secretion, Kang's group reported that BM metastatic breast cancer cells induce osteoclastogenesis exclusively by directly activating Notch signaling on OCLs through tumor cell-derived Jagged [34]. Therefore the mechanism here described is unique. Nonetheless, the exploitation of the RANKL-based mechanism by MM cells should not surprise. Indeed, the engagement of RANK by RANKL in pre-OCL was previously reported as key for physiological OCL differentiation, since it resulted in NF-κB and Notch activation and the subsequent increase in the expression of NFAT1c, a master regulator of osteoclastogenesis [28, 45].

We further investigated the molecular events triggered by RANKL in OCL progenitors during differentiation (illustrated in figure 8). One issue regarded the controversy on the specific role of the Notch isoforms in the osteoclastogenic process. Choi and colleagues [46] suggested that RANKL-induced OCL differentiation is promoted by Notch1 intracellular domain, whereas Bai et al. described Notch1 negative effect on osteoclastogenesis [27]. Our data support the concepts that Notch1 activity is neither necessary, since it was downregulated during RANKL-induced Raw264.7 cells differentiation, nor sufficient to induce osteoclastogenesis, due to the observed lack of differentiation of ICN1-transfected Raw264.7 cells. Oppositely, the RANKL-dependent increase of Notch2 during Raw264.7 cells differentiation confirmed that this isoform is essential as previously reported by Fukushima et al. [28]. Nonetheless, differently from these authors, who reported that Notch2 boosted OCL differentiation induced by RANKL, our results indicated that Notch2 forced expression alone was sufficient to stimulate osteoclastogenesis by promoting an autonomous secretion of RANKL in Raw264.7 cells.

The other relevant information generated by this work concerns a new form of cooperation of Notch with the NF-kB pathway during OCL differentiation. The evidences that RANK increase during Raw264.7 cell differentiation can be hampered by Notch inhibition indicates that Notch signaling activation, observed during osteoclastogenesis, increases pre-osteoclast responsiveness to RANKL by promoting the expression of its receptor RANK.

The relevance of the two dysregulated Jagged ligands in the MM cell osteoclastogenic ability, makes them promising targets for a Notch inhibitory approach aiming to counteract the MM-related osteoclastogenesis and co-morbidities. Indeed, we observed that Jagged1 and Jagged2 silencing in U266 cells decreased Notch activity along with the ability to induce OCL differentiation via a direct or indirect (RANKL-mediated) activation of Notch activity on Raw264.7 cells. Moreover, we demonstrated that even the expression of RANKL induced by interaction with stromal cells in naturally low RANKL-expressing cells, such as OPM2, could be inhibited by J1/J2 silencing. Moreover J1/J2 silencing can effectively inhibit the autonomously activated Notch signaling, whose promoting effects on MM growth and survival have been widely illustrated in the recent years [3, 4, 23, 24, 26, 38, 41].

A Notch-directed approach based on Jagged inhibition could be more selective and safe if compared with GSIs which causes gut toxicity due to the contemporaneous inhibition of all the Notch isoforms [3]. The redundancy of Notch ligands and the efficacy of Jagged1 and Jagged2 inhibition in reducing the excessive Notch signaling in MM cells, may provide the rational for an effective and safer Notch-directed approach to target MM patients bone disease and the associated co-morbidities, including increase in tumor burden [10], angiogenesis [12], drug resistance [35, 36] and inhibition of immune response [3, 11].

## MATERIALS AND METHODS

### Cells and treatments

All cells were maintained in 5% CO_2_ atmosphere. The murine cell lines Raw264.7 and NIH3T3 and the human BMSC line HS5 were cultured in complete DMEM medium with 10% heat inactivated FBS, the human MM cell lines U266 and OPM2 in complete RPMI1640 with 10% heat inactivated FBS. Following reconstitution in DMSO, DAPT (Sigma Aldrich, Germany) was administered to cells at a final concentration of 50μM. Recombinant mouse RANKL (mRANKL, Peprotech, USA) was used at the final concentration of 50ng/ml. Anti-RANKL neutralizing antibody (Peprotech, USA) was used at the final concentration of 0.10μg/ml.

### Osteoclastogenesis assays

OCL differentiation was induced as reported in each experiment. On the day of harvest, cells were fixed on the culture plates with citrate-acetone solution and stained for TRAP (Sigma-Aldrich). Osteoclasts were identified and enumerated under light microscopy as TRAP^+^ cells with ≥3 nuclei. Further details on treatments and experimental procedures are provided in Supplemental Information.

### Bone resorption assay

Raw264.7 were cultured on Osteo Assay Surface 24-wells plates (Corning) under differentiation conditions. After 7-10 days of culture, the plates were washed in 5% sodium hypochlorite solution to remove the cells. The resorbed areas on the plates were captured with EVOS fl microscope and measured by using the Wimasis image analysis software (GmbH) to process 20x pictures covering the whole well surface.

### qRT-PCR

Total RNA from cell lines was isolated, cDNA was prepared and Quantitative PCR (qPCR) was performed as previously described [4]. Total mRNA for qRT-PCR on primary human cells was isolated using the miRNeasy kit (Qiagen). Primers are reported in Table1 (Supplemental Information).

### ELISA Assay

Flat-bottom 96-well polycarbonate plates were coated at 4°C overnight with 50 μL/well cell culture supernatants diluted 1:1 in carbonate coating buffer (0.1 M Na_2_CO_3_, 0.1 M NaHCO_3_, pH=9.5). Standard curves were obtained with serial dilutions of purified recombinant human RANKL (Merck-Millipore) or recombinant mouse RANKL (Peprotech). After blocking with PBS supplemented with 1% W/V BSA, plates were incubated with biotin-conjugated goat anti-human RANKL (Merck-Millipore) or rabbit anti-mouse RANKL (Peprotech, USA) for 1 h at RT. Then, plates were washed with PBS-0.025% V/V Tween-20 and incubated at RT with Streptavidin-HRP-labeled secondary antibody (Invitrogen) or with a mouse anti-rabbit secondary antibody (Santa Cruz Biotechnology, Inc) for 30 min. The plates were washed, then the TMB substrate (Thermo Scientific, Inc) was added, and signal was measured using a microplate reader. All samples were run in triplicates.

### RNAi Assay

Two stealth small interference RNA (siRNA) molecules targeting Jagged1 (CGCGACGAGUGUGACACAUACUUCA, UGAAGUAUGUGUCACACUCGUCGCG) and Jagged2 (GCCUUGCUACAAUGGUGGCAUUCUGU, ACAGAUGCCACCAUUGUAGCAAGGC) and a negative control were purchased from Invitrogen. Select RNAi^TM^ siRNA system (Invitrogen) was used according to the manufacturer's guidelines.

### Transfections

Intracellular Notch1 (ICN1) and Notch2 (ICN2) constructs were as described (47, 48). One hundred microliters of Raw264.7 cell suspension (10^7^/mL) in RPMI1640 w/o antibiotics were mixed with 5 μg DNA, transferred into a 2.0 mm-gap cuvette (BTX, MA, USA), electroporated at 250 V and 950 μF and cultured for 3 days in a 6-well plate.

### Gene expression profiling

BM highly purified (CD138 ≥ 90%) PC samples from 55 newly diagnosed MM patients were profiled on the GeneChip Human Gene 1.0 ST array (Affymetrix, Santa Clara, CA). Gene expression profiling data were generated as previously described [31]. The Institutional Review Board of Fondazione IRCCS Policlinico Ca' Granda, Milano, Italy, approved the design of this study. Written informed consent in accordance with the Declaration of Helsinki was obtained.

## SUPPLEMENTARY INFORMATION FIGURES AND TABLE


