# Supplementary material for: Notch signaling drives multiple myeloma induced osteoclastogenesis
Source: Oncotarget. 2014 Jun 9;5(21):10393–406. doi: 10.18632/oncotarget.2084 (PMC4279381; doi:10.18632/oncotarget.2084)
Supplement: Supplementary file 1 [file oncotarget-05-10393-s001.doc]

**SupplementaRy Information**

**Notch signaling drives multiple myeloma induced osteoclastogenesis**

Michela Colombo, Katja Thümmler, Leonardo Mirandola, Silvia Garavelli, Katia Todoerti, Luana Apicella, Elisa Lazzari, Marialuigia Lancellotti, Natalia Platonova, Moeed Akbar, Maurizio Chiriva-Internati, Richard Soutar, Antonino Neri, Carl S. Goodyear and Raffaella Chiaramonte

**INVENTORY**

Supplemental Information contains the Supplemental Data (2 figures) and Supplemental experimental Procedures.

Supplemental Data:

Figure S1 is related to Figures 1.

Figure S2 is related to Figure 3.

**
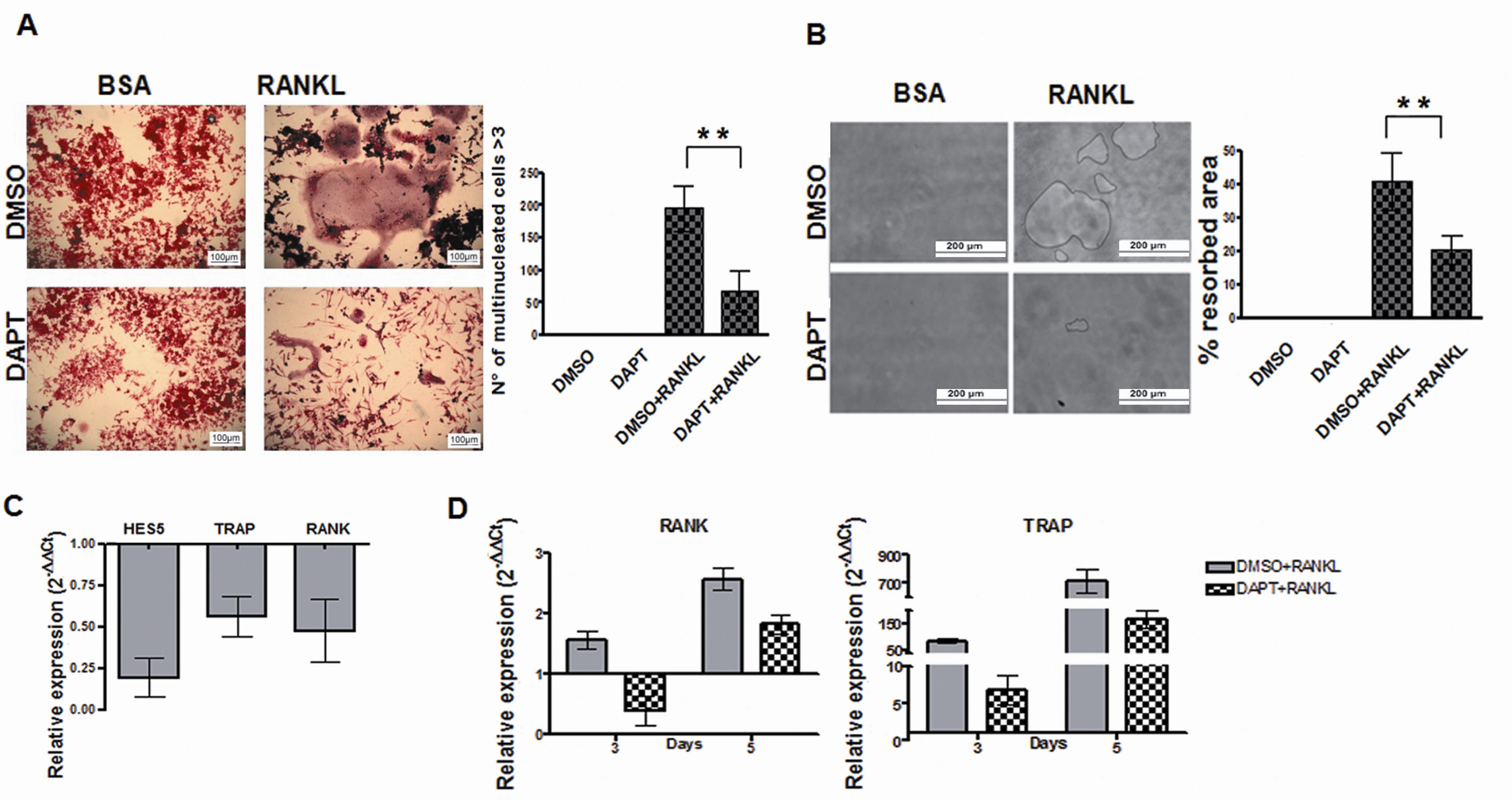
**

**Figure 1. The Notch pathway is necessary for osteoclasts differentiation and activity**.

To assess DAPT effect on OCL differentiation in Raw264.7 cells, we performed:

(A) TRAP staining/enumeration of multinucleated cells

(B) Pit formation Assay on Raw264.7 cells treated with or without DAPT.

Mean values ± standard deviations (SD) are calculated out of 3 independent experiments. Statistical analysis by ANOVA and Tukey post-test; **= p <0.01.

(C-D) qRT-PCR analysis of TRAP and RANK genes in (C) Raw264.7 cells treated with DAPT for 72 hours. or (D) with mRANKL and DAPT for 3, 5 days. The relative gene expression of TRAP and RANK was calculated by the 2−ΔΔCt formula by normalizing the expression level to GAPDH and comparing the normalized gene expression value of DAPT treated Raw264.7 cells to control cells (DMSO treated cells=1). Two-tailed t-test confirmed statistically significant differences in the expression levels of the target genes (p<0.01).

**Figure S2. Notch2 promotes RANKL expression in OCL precursors and promotes differentiation.**

1. Western blot on Raw264.7 cells after 48h from the electroporation with two vectors expressing the active forms of Notch1 and Notch2. Pictures are representative of at least three experiments with similar results.
2. qRT-PCR shows that Notch2 overxpression in Raw264.7 cells is able to promote differentiation (as confirmed by the increase in TRAP levels) and to upregulate RANKL expression. The increased HES5 gene expression confirms the activation of Notch signaling. Gene expression is normalized to GAPDH. Data are presented as relative gene expression in Notch2-transfected cells compared to Mock transfected cells (Mock =1), and are calculated by the 2−ΔΔCt formula. Error bars represent SD calculated out of 3 independent experiments.
3. TRAP staining of Raw264.7 cells induced to differentiate for 7 days in the presence of CM from Notch1- and Notch2-transfected Raw264.7 cells. The osteclastogenic effect of RANKL released in the CM by Notch2-transfected cells was confirmed by the inhibition of OCL differentiation through the addition of RANKL neutralizing antibody in the CM. Picture are representative of at least 3 experiments with similar results.

**Supplemental experimental Procedures**

# Osteoclast differentiation from RAW264.7 cells

Raw264.7 cells were seeded on a 24-well plate at a density of 1×10^4^ cells/well. Cells were treated for 5 days with mRANKL with or without DAPT.

For co-culture experiments Raw264.7 and U266 cells were seeded on a 6-well plate at a density of 1×10^4^ cells/well (about 8×10^3^ Raw264.7 and 2×10^3^ U266) and cultured for 7days in the presence/absence of DAPT.

For experiments with the different conditioned media, Raw264.7 cells were seeded on a 6-well plate at a density of 1×10^4^ cells/well and allowed to adhere for 3hours. Then, medium was replaced with 4/5 of DMEM and 1/5 of CM from U266 cells, U266 cells transfected with J1/J2- or Scr-siRNAs, OPM2 +/- NIH3T3 co-culture system or Raw264.7 cells transfected with ICN1 or ICN2 expressing plasmids. CM were replaced every 2-3 days. Cells were cultured for the time reported in Results.

For experiments of OCL differentiation induced by Jagged1 peptide, Raw264.7 cells (2x10^5^ cells/well) were cultured for 7 days in a 12-well plate in the presence of 30ng/ml mRANKL and Jagged1 peptide (AnaSpec, Inc) dissolved in the culture medium (0.5μg/ml) or immobilized on the well surface by coating with 0.5μg/ml Jagged1 in PBS-0.1% gelatin.

On the day of harvest, cells were fixed on the culture plates with citrate-acetone solution and stained for TRAP (Sigma-Aldrich). Osteoclasts were identified and enumerated under light microscopy by the presence of ≥3 nuclei by using an Olympus IX71 microscope equipped with a XC10 camera.

**Osteoclast differentiation from primary human CD14^+^ monocytes**

1x10^6^ /ml primary monocytes were cultured in complete α-MEM with 10% FBS and 25ng/ml human m-CSF (PeproTec) with or without 100ng/ml human soluble RANKL (PeproTec) for 8 days in the presence (25μM) or absence (DMSO) of DAPT. Media and stimuli were changed every third day.

For co-culture experiments, purified primary myeloma cells (1x10^6^/ml) were co-cultured with pre-osteoclasts (1x10^6^/ml; treated with 25ng/ml human m-CSF (PeproTec) for 12h) for 7 days in complete α-MEM with (25μM) or without (DMSO) of DAPT. Media and stimuli were changed every third day.

On the day of harvest, osteoclasts were identified and enumerated as reported above by using Olympus IX51 microscope (Olympus, UK) with Olympus TL4 Lamp (Olympus, UK). Images were captured using Cell^D Software (Olympus, UK).

**RT-qPCR Primers**

Here are reported all the primers used for the RT-qPCR analysis.

| **RT-qPCR primers** | **Forward Primer 5’-3’** | **Reverse Primer 5’-3’** |
| --- | --- | --- |
| **mGAPDH** | TTGGCCGTATTGGGCGCCTG | CACCCTTCAAGTGGGCCCCG |
| **mHES5** | GGCTCACCCCAGCCCGTAGA | TCGTGCCCACATGCACCCAC |
| **mCXCR4** | AACCACCACGGCTGTAGAGCGA | TCCCGGAAGCAGGGTTCCTTGT |
| **mTRAP** | ACCGTGCCCTTCGCAACATCC | GACAGCTGAGTGCGGGCCAC |
| **mRANK** | TGCCCTGTGGCCCCGATGAG | TGGTAGCCAGCCGTGCAAGC |
| **mRANKL** | CCCAGCGAGGCAAGCCTGAG | TGCCGAAAGCAAATGTTGGCG |
| **mNOTCH1** | ACCGGAGTGGACGGGTCAGT | TGTGCGCCCATGCGGACATT |
| **mNOTCH2** | CTTGCTTGTGCCCCGTGGGT | GCCCGAGTGCTGGCACAAGT |
| **hGAPDH** | ACAGTCAGCCGCATCTTCTT | AATGGAGGGGTCATTGATGG |
| **hHPRT1** | GTAGCCCTCTGTGTGCTCAA | TTTATGTCCCCTGTTGACTGGT |
| **h/m18s** | GTAACCCGTTGAACCCCATT | CCATCCAATCGGTAGTAGCG |
| **hHES6** | ATGAGGACGGCTGGGAGA | ACCGTCAGCTCCAGCACTT |
| **hJAG1** | TTCGCCTGGCCGAGGTCCTAT | GCCCGTGTTCTGCTTCAGCGT |
| **hJAG2** | CCGGCCCCGCAACGACTTTT | CCTCCCTTGCCAGCCGTAGC |
| **hRANKL** | AAGGAGCTGTGCAAAAGGAA | CGAAAGCAAATGTTGGCATA |
| **hB2M** | TAGCTGTGCTCGCGCTACT | TCTCTGCTGGATGACGTGAG |
| **hRANK** | gctgtaacaaatgtgaaccagg | gccttgcctgtaTcacaaact |
| **hCathepsinK** | ttggaagggagttggtgtg | tgggtggagagaagcaaagt |

**Tab1. Primer sequences**.

## Western Blot Analysis

Whole cell extracts were prepared using a RIPA lysis buffer containing 50mM Tris-HCl, pH 7.4, 150mM NaCl, 1% Triton X-100, 1% sodium deoxycholate, 0.1% SDS, 1mM EGTA, 1mM EDTA and the protease inhibitors, 50mM NaF, 1mM phenylmethylsulfonyl fluoride (PMSF), 1 mM Na_3_VO_4_, 2μg/ml aprotinin, 2μg/ml leupeptin. After incubation on ice for 15 min, the lysate was clarified by centrifugation for 10 min at 4°C. Protein concentration was determined using the Bradford assay (Bio-Rad Laboratories). Protein samples (50–70 μg) were loaded and run on 8% denaturing SDS-PAGE gels, transferred to a nitrocellulose membrane (Hybond-ECL, Amersham Bioscience), and blocked with 5% screamed milk in TBS-T (20 mM Tris, pH 7.5, 150 mM NaCl, 0.05% Tween-20). The membrane was incubated o.n. at 4°C with the indicated primary antibodies as follows: cleaved-Notch1 Val1744 (Cell Signaling Technologies) 1:1000, Notch2 intracellular domain antibody-cleaved Asp1733 (Abcam) 1:1000, βActin antibody (Santa-Cruz Biotechnology), 1:1000. Following washes, the filter was incubated with HRP-conjugated species-specific secondary antibodies (Santa-Cruz Biotechnology). Proteins were visualized with ECL reagents (Promega) according to the manufacturer’s instructions.

**ELISA assay**

Flat-bottom 96-well polycarbonate plates were coated at 4°C overnight with 50 µl/well cell culture supernatants diluted 1:1 in carbonate coating buffer (0.1 M Na2CO3, 0.1 M NaHCO3, pH=9.5). Standard curves were obtained with serial dilutions of purified recombinant human RANKL (Merck-Millipore) or recombinant mouse RANKL (Peprotech). After blocking with PBS supplemented with 1% W/V BSA, plates were incubated with biotin-conjugated goat anti-human RANKL (Merck-Millipore) or rabbit anti-mouse RANKL (Peprotech, USA) for 1 h at RT. Then, plates were washed with PBS- 0.025% V/V Tween-20 and incubated at RT with Streptavidin-HRP-labeled secondary antibody (Invitrogen) or with a mouse anti-rabbit secondary antibody (Santa Cruz Biotechnology, Inc) for 30 min. The plates were washed, then the HRP-substrate was added, and signal was measured using a microplate reader. All samples were run in triplicates.

**Flow cytometry**

RANKL expression in OPM2/HS5 co-cultures was evaluated by flow cytometry. Prior to co-cultivating the two populations, HS5 cells were stained with the fluorescent dye PKH26 (Sigma-Aldrich). RANKL expression in tumor cells was assessed in PKH26- cells. For RANKL detection, cells were fixed with 4% formaldehyde, permeabilized in 0.5% saponin and stained with anti-human RANKL antibody (Abcam Plc, UK) or isotype matched control. After 1h incubation at 4 °C in the dark, cells were washed and the Alexa 488-conjugated secondary antibody was added (anti-rabbit antibody, Life Technology Inc). Cells were processed using the Cytomics FC500 software (Beckman Coulter s.r.l., Milano, Italy).
